# Diagnosis of Tubercular Meningitis by Means of the Ophthalmoscope

**Published:** 1869-08

**Authors:** 


### Diagnosis of Tubercular Meningitis by means of the Ophthalmoscope.

In the *Jahrbuch fur Kinderheilkunde,* of March 10th, 1869, Dr. Frankel, of Berlin, has an article on the coincidence of tuberculosis of the choroid coat of the eye, with general miliary tuberculosis of children. The diagnosis of tubercular meningitis is especially difficult in the earlier stages, and would probably always have remained so if Cohnheim had not discovered by numerous careful post-mortem examinations that tubercular deposits on the choroid coat are an almost constant attendant of miliary tuberculosis. Previously to Cohnheim's times this coincidence had been regarded as an occasional and accidental pathological curiosity; he proved that it was of constant occurrence. Von Graefe at once saw the importance of this fact in a diagnostic point of view and quickly diagnosed a case. To Dr. Frankel belongs the credit of detecting the next two cases.

The appearance of choroid-tubercles in the fundus of the eye is very characteristic and easily understood if one reflects that they (the tubercles) originate in the stoma of the choroid, and only project into the eye after pushing some of the pigment aside. Thus arise small, roundish, white spots which are continuous with the normal tissues by means of adherent, or grown-together edges. The whole of the rest of the background of the eye appears normal with the exception of these spots and a more or less considerable congestion of the retina. If a blood-vessel happens to run over one of these tubercles, it will be somewhat pushed aside as the tubercle grows larger. In one instance Frankel noticed a rapid increase of this changed course of a blood-vessel, and concluded that the tubercle was growing rapidly.

In two cases he noticed an old tubercle in the centre, and fresher and brighter tubercular spots around it. Choroid tubercles are most frequently found in the neighborhood of the pupilla, and this renders their discovery with the opthalmoscope comparatively easy. They are so peculiar and characteristic in appearance that a mere momentary glimpse of them is sufficient to establish a diagnosis. They do not cause any disturbances of vision, at first; neither spots or sparks before the eyes, nor intolerance of light. The patients, if old enough, can read and write, and see perfectly. In short they cannot be suspected or detected except by the opthalmoscope; but if once seen, the physician may be very certain that his patient will die sooner or later of tubercular meningitis, or general miliary tubercular deposits in many organs.

A diagnosis is especially important between simple pneumonia in children when attended with delirium, coma, and other cerebral symptoms, and miliary tuberculosis of the lungs when associated with tubercular meningitis. The symptoms of the two diseases are very much alike, but the latter is an almost necessarily fatal disease, while the former is among the most curable. The only distinguishing marks as yet have been that the heat of the skin never rises so high in the tubercular disease as it does in pneumonia; neither is the elevation of the temperature so continuous; nor does it fall so rapidly and decidedly as it does on the critical days of convalescence from pure and frank pneumonia. In cases in which we are in doubt between cerebral and pneumonia, and tubercular meningitis associated with miliary tubercles in the lungs and other organs, if the heat of the skin is very great, and no sign of the tubercles can be detected in the choroid, we may safely conclude that we are dealing with the milder disease, although the symptoms may be much more severe. If this diagnostic sign had been known before, it is almost certain that so many cases of supposed recovery from tubercular meningitis would not have been recorded.—*Med. Gazette.*

